# Conversion of dissolved phosphorus in runoff by ferric sulfate to a form less available to algae: Field performance and cost assessment

**DOI:** 10.1007/s13280-014-0622-8

**Published:** 2015-02-15

**Authors:** Risto Uusitalo, Aaro Närvänen, Antti Kaseva, Aino Launto-Tiuttu, Janne Heikkinen, Päivi Joki-Heiskala, Kimmo Rasa, Tapio Salo

**Affiliations:** 1 Natural Resources Institute Finland, 31600 Jokioinen, Finland; 2 Turku University of Applied Sciences, Sepänkatu 1, 20700 Turku, Finland; 3 TEHO Plus Project, Varsinais-Suomi Centre for Economic Development, Transport and the Environment, PL 523, 20101 Turku, Finland; 4 Paimionjoki-yhdistys ry, PL 41, 31401 Somero, Finland

**Keywords:** Phosphorus, Ferric sulfate, Eutrophication, Mitigation

## Abstract

Conversion of dissolved P by ferric sulfate into a particulate form sparingly available to algae was studied in 15 ditches in Finland using stand-alone dispensers for ferric sulfate administration. Ferric sulfate typically converted 60–70 % of dissolved P into iron-associated form, a process which required 250–650 kg per kg dissolved P. Mean cost was 160 EUR per kg P converted (range 20–400 EUR kg^−1^). The costs were lowest at sites characterized by high dissolved P concentrations and small catchment area. At best, the treatment was efficient and cost-effective, but to limit the costs and the risks, ferric sulfate dispensers should only be installed in small critical source areas.

## Introduction

To combat eutrophication of surface waters, the EU Water Framework Directive (Directive 2000/60/EC) prescribes restoration or enhancement of the chemical and ecological status of water bodies. Because agriculture is currently considered a major contributor to phosphorus (P) loading of surface waters (e.g., HELCOM 2009), agricultural sources need to be included in P mitigation plans.

The nutrient loads from agriculture are affected by enterprise type and intensity. A site with high soil P concentrations and a hydrological connection to a stream through a ditch network or tile drainage system will contribute to the total P loads in a body of water to much greater extent than reflected by its proportion of catchment area. Relatively small (hot-spot) areas develop, for example, in places that receive continuous inputs of manure, such as feedlots, around dairy houses and around water stations in pastures (e.g., Weld et al. 2001; Page et al. 2005).

Dissolved P concentrations in runoff tend to be particularly elevated as a result of long-term P accumulation in soil. The dissolved P pool is generally considered to be totally available for biological utilization (Ekholm and Krogerus 2003), whereas much of the P present as particulate P (PP) may be practically inert in the short term (Sonzogni et al. 1982). Particulate P can partly enter the biological cycle under special circumstances (Lehtoranta et al. 2015), but when aiming to limit the amount of P readily available to aquatic algae, dissolved P is logically the first priority for remedial actions because it can immediately trigger algal growth in P-limited waters.

Management of soil P stocks, limiting mineral fertiliser and manure/slurry inputs and fencing off streams from livestock have been identified as the most cost-effective ways to reduce losses of dissolved P, whereas the use of soil amendments and edge-of-field measures is associated with distinctly higher abatement costs (McDowell and Nash 2012). However, when treating runoff from hot-spot areas the use of higher-cost measures can be justified, because P input management has a long lag time between implementation and effect. Potential options for mitigating hot-spot P losses include decreasing P mobilization or intercepting transported P using chemicals or a reactive medium as a soil amendment, as an edge-of-field P-trap, or in ditches that connect to larger waterways.

In particular, the use of solid by-products as P filter materials has attracted strong interest in recent years (Brooks et al. 2000; Vohla et al. 2011; Buda et al. 2012). Although most published studies involving P sorbents are restricted to P retention measurements in the laboratory, some field data on the use of different types of materials are also available (McDowell et al. 2007; Penn et al. 2007; Shipitalo et al. 2012). At best, P sorbents can substantially decrease P concentrations and loads to adjacent waterways. However, in regions with a long winter period associated with soil frost, snow accumulation and rapid snowmelt that carries a substantial proportion of the annual P load to watercourses within a short period (Rekolainen 1989), such filters would be still frozen during the time of the high P losses in early spring.

Another option could be to decrease bioavailability of P by adding chemicals that react with dissolved P to runoff water, making the P less readily available for freshwater algae and bacteria (Neufeld and Thodos 1969; Närvänen et al. 2008). Metal salts such as ferric or aluminum sulfates or chlorides, which are widely used in water and wastewater purification, could be used for this purpose. They can be regarded as a proven option in engineered treatment facilities that allow optimization of their use efficiency, but when used to treat agricultural runoff they would be applied in very different environmental settings.

We examined the use of a simple type of ferric sulfate dispenser for reducing the concentrations of dissolved P in stream runoff, using as test sites 15 agricultural ditches in SW Finland. The objective was to evaluate the P reduction effect in variable field conditions, the practicality of the method and the cost of converting dissolved P to a sparingly available form. The starting hypothesis was that the method is suitable for use in early spring, when the soil is frozen and natural P retention processes are inactive.

## Materials and methods

### Chemicals

The chemical additive used for stripping soluble P from water was ferric sulfate, Fe_2_(SO_4_)_3_ (Chemical Abstracts Service identifier 10028-22-5). A commercial ferric sulfate product with the trade name Ferix-3 was obtained from Kemira Kemwater Oy (Helsinki, Finland). Ferix-3 is used in wastewater treatment plants and in tap water purification to precipitate solids, organic matter and dissolved P. It has an iron content of about 20 %, with the iron entirely in oxidation state +3. The chemical is soluble in water, so solutions containing about 40 % (by weight) are stable. Ferric sulfate falls into the ‘irritant’ chemical hazard class, but has a pH in aqueous solution of less than 2. Ferix-3 is manufactured as granules with typical mean diameter 2 mm (96 % between 0.2 and 5 mm) and volume weight 1.2 kg L^−1^.

### Dispenser device

**Fig. 1:**
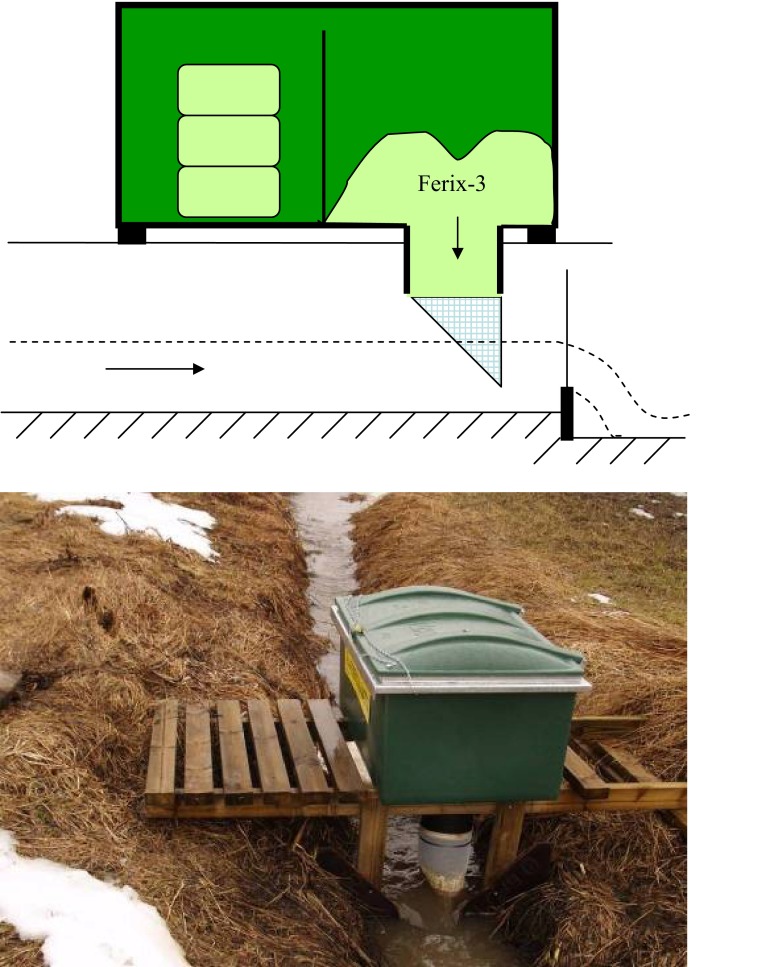
Schematic diagram of the ferric sulfate dispenser used in the study (*upper*) and photograph of a unit in work (*lower*). Photograph: Aaro Närvänen

For dispensing the Ferix-3 into stream water, a dispenser developed previously at MTT by Aaro Närvänen was used (Närvänen et al. 2008). A schematic diagram of the device and a unit in operation are shown in Fig. 1. The dispenser consists of a container for chemicals (150–600 L capacity hopper) that has a 200-mm polyethylene pipe attached to a hole in the base and a cone shaped polyester netting attached to the lower end of the polyethylene pipe. The chemical granules are fed from the hopper into the pipe by gravity and down into the netting cone, which is partly submerged during flow events. The proportion of the cone that is submerged is controlled by the water level above a v-notch weir. As flow increases, the v-notch weir retains more water and the more of the netting cone is submerged, thus increasing chemical dose.

Dosing of the chemical can be fine-tuned by changing the angle of the v-notch weir. In this study the v-notch weir was occasionally set at an angle of 90°, but in most cases it was set at 120°. The latter is usually sufficient when the ratio of administered chemical to ditch water is 1 kg to approximately 50 m^3^ water. This dose has been found in earlier tests to cause precipitation of more than 80 % of the dissolved P, but not precipitation of most suspended solids (Närvänen et al. 2008). Precipitation of suspended solids was not desired as it would result in the production of large volumes of sludge and hence aesthetic issues and possibly backed-up ditches.

### Test sites and sampling

#### The Nautela site

The dispenser was tested in 15 agricultural ditches in SW Finland. A continuously operated unit was used at Nautela, Lieto (60°33′N 22°27′E), near the city of Turku in SW Finland, and is hereafter referred to as the Nautela unit. The catchment area for that ditch is about 60 ha and the proportion of arable fields in the catchment is 63 %. The main soil types are silty and loamy clays and the fields have a typical slope of <3 %. The fields were under cereal production during the tests.

Along the ditch there are two small ponds, created by bottom dams, about 100 m from each other. A ferric sulfate dispenser equipped with a 350-L chemical hopper and a 120°-angle v-notch weir was installed in the ditch section between the ponds, where there was a spot with a steep enough gradient for weir construction.

In the vicinity of the v-notch weir, a water level sensor (Keller DCX-22; Keller AG, Winterthur, Switzerland) was installed to enable discharge calculations. YSI 6920 series multiparameter (Xylem Inc., NY, USA) and Scan Nitrolyser (Scan Messtechnik GmbH, Vienna, Austria) sensors were placed in two spots in the ditch, before and after the dispenser unit, for monitoring water quality parameters (pH, turbidity, etc.). The sensors were set to record data every 30 min. The water level monitor records were checked by comparing with manual water level readings in the v-notch weir when the site was visited (weekly), and the multiparameter sensors were regularly calibrated and cleaned.

For chemical analyses, a total of 56 pairs of water samples (one sample upstream and one downstream of the ferric sulfate dispenser unit making a pair) were taken manually at Nautela from April 2011 to October 2012. Manual grab sampling was carried out at the same spots where the sensors were located, generally once a week but less frequently during dry spells. The ditch froze over during winter, so the results cover the ice-free season from spring floods to the onset of frost in autumn for the years 2011 and 2012.

For estimation of P fluxes at Nautela, the concentrations measured in grab water samples were first multiplied by the water volume recorded during the 24-h period around the sampling events (12 h before and after sampling), assuming that P concentrations measured in the grab samples represented the mean concentrations of the 24-h periods in question. The P masses obtained by this calculation were then added up and the values extrapolated to the rest of the operating period. Extrapolation was done by multiplying the P mass of sampling days by a scaling factor that was calculated as: recorded water flow during the whole study period divided by the flow recorded during the 24-h time slot of the water sampling dates. These calculations of P fluxes were performed separately for upstream and downstream positions of the dispenser unit and the conversion of dissolved P to iron-associated form was calculated as their difference.

#### Other test sites

Other ferric sulfate dispenser units were operated in 10 ditches draining into Lake Nuutajärvi (61°02′N 23°27′E) in Urjala community (sites N1–N10; Table 1), one site in Tammela community (60°48′N 23°45′E; site T1) and three sites (P1–P3) by the river Paimionjoki in Somero community (60°48′N 23°45′E). The ditches selected for the tests had varying catchment size (0.2–160 ha) and nutrient sources, as some were surrounded by animal husbandry and some by only arable fields (Table 1).

**Table 1:** Catchment area and agricultural activities at the 15 study sites. Approximate site locations can be viewed via the URLs (created 13 Nov 2014)

Site	Catchment (ha)	Source of the loading	Google map URL
Nautela	60	Fields	http://goo.gl/dBbbEZ
N1	<5	Cowshed, exercise yards, fields, forest	http://goo.gl/0LPFFY
N2	50	Cowshed, exercise yards, fields, forest	Approximate for N1–N10
N3	1	Cowshed yard	
N4	140	Forest, pasture	
N5	<5	Cowshed, exercise yards	
N6	50	Fields	
N7	20	Fields	
N8	5	Horse stable yard	
N9	>100	Fields and forest, about equal shares	
N10	0.2	Horse stable yard	
T1	>5	Manure pit, silage bay, farmhouse	http://goo.gl/PTVIbX
P1	160	Fields (30 %) and forest (70 %)	http://goo.gl/H0ClzJ
P2	20	Piggery, fields receiving pig slurry	Approximate for P1–P3
P3	25	Fields, cowshed, piggery, forest (20 %)	

Because of minimal or non-existent flow in the vast majority of the ditches during the summer and during several months of ground frost in winter, the dispenser units were operated and water sampling was carried out over an interval of some days as long as flow occurred. The sites N1–N10 were sampled during 22 March–11 April in 2012 and during 15–26 April in 2013, with a total of 46 sample pairs being collected. Site T1 was operated from autumn 2010 to onset of frost in 2012, but only five sample pairs in total were obtained as a result of snowmelt and storm runoff overwhelming the dispenser unit. For sites P1–P3, the tests in 2012 started on 8 October and ran until 5 November, when frost set in. In 2013, operation and sampling at sites P1–P3 commenced at the start of spring flow, on 15 April, and ended on 3 June. A third test period at sites P1–P3 started on 20 October 2013 and ended on 12 November. A total of 66 water sample pairs were obtained from these three sites.

For the 14 sites without flow measurement instrumentation, flow was recorded on sampling visits and the fluxes of dissolved P were estimated by assuming that both the flows and concentrations increased or decreased linearly between the recorded values. This assumption most likely resulted in inaccurate estimates of water and P flows, but the sites were studied for short periods and visited at intervals of a few days, so the errors were not expected to be biased in one direction or another. The mass of converted dissolved P due to ferric sulfate application was obtained by using an average percentage change due to the treatment at a given site.

#### Water analyses

Chemical analyses of P concentrations were conducted on both unfiltered and filtered (0.2 µm pore size membrane filters; Nuclepore, Whatman, Maidstone, UK) water samples. The unfiltered samples were pre-treated by digestion with peroxodisulfate in an autoclave for determination of total P (TP) concentrations. The filtered subsamples were analyzed for dissolved P, either without digestion [i.e., dissolved reactive P (DRP) for sites N1–N10 and T1] or after digestion [i.e., total dissolved P (TDP), for Nautela and sites P1–P3].

The reason for conducting different dissolved P analyses (DRP or TDP) at different sites was a result of using two separate laboratories, one at MTT Agrifood Research Finland and the other a commercial certified laboratory. It eventually emerged that there was a misunderstanding in communication with the commercial laboratory employed to analyze the samples from Nautela and the sites P1–P3. The digestion step in TDP analysis would liberate any P attached to colloidal mineral and organic matter that passed through the 0.2 µm filter membrane used, and therefore the results may represent slightly different P pools in the samples analyzed at the two laboratories. However, if ferric sulfate treatment does remove dissolved P from runoff water, the results would still point in the same direction.

A LaChat QuickChem 8000 analyzer (LaChat Instruments, Loveland, CO, USA) and a Beckman DU-640i analyzer (Beckman Coulter, Inc., Brea, CA, USA) were employed in P analyses. Limit of detection in both laboratories was 2–3 µg L^−1^, and measurement uncertainties was 15–20 % for <25 µg L^−1^ P concentrations and 10–15 % for higher concentrations. Glass electrodes were used to measure the pH of water samples.

#### Statistical analyses

Statistical significance of differences in median concentrations was tested as paired comparisons using the Wilcoxon signed ranks test with two-tailed probability distributions. This non-parametric test was chosen because of non-normal distributions of sample. For those test sites that had a number of observations exceeding 20, each site was tested separately. However, sites N1–N10 and T1 had few (7 at maximum) observations per site, so the data for these sites were pooled into ‘Low DRP’ and ‘High DRP’ classes (the division was set at median DRP ≥ 100 µg L^−1^ in incoming water). After pooling, the Low DRP group had 31 observations and the High DRP group 19 observations.

## Results

### Tests at the Nautela site

**Fig. 2:**
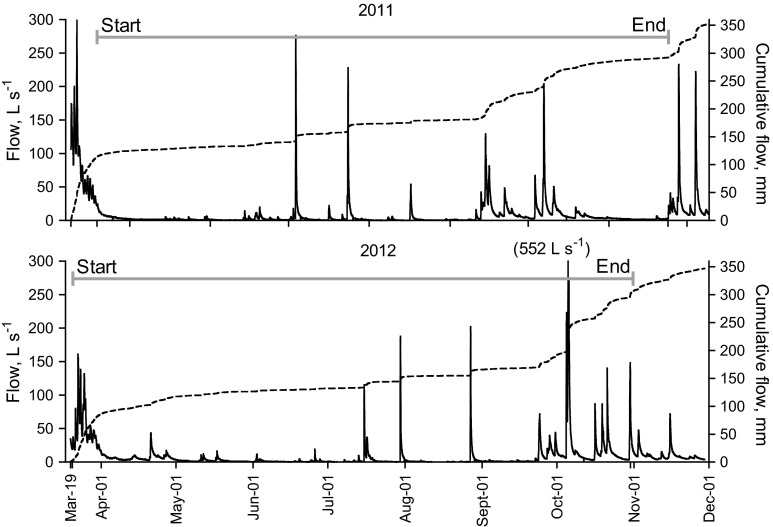
Flow events (*solid line* left *y*-axis) and cumulative flow (*dotted line* right *y*-axis) measured at the Nautela site during 2011 and 2012. The *horizontal gray lines* indicate the start and end of chemical dosing and sampling in the 2 years. The 552 L s^−1^ in *brackets* in the lower graph indicates the maximum flow peak (off the left *y*-axis scale) after two major storm events on 4 and 5 October 2012

Flow fluctuations in the Nautela ditch were considerable during the study, varying from <0.1 L s^−1^ in dry spells to up to more than 500 L s^−1^ after heavy rain. Snowmelt in spring was the most intensive flow period during both study years. In 2011, up to about a third of total flow occurred during about 2 weeks in April, while in 2012, 25 % of all flow occurred over a few days in late March–early April (Fig. 2). In 2011, the first snowmelt peak in April, with 75 mm cumulative flow, occurred before ferric sulfate treatment was started, while in late autumn of the same year chemical treatment ended before storm flows of 60 mm (Fig. 2). Due to these timing mismatches and occasional chemical run-out events in 2011, only about 180 mm of the 350 mm recorded cumulative flow at this site (51 %) was treated with ferric sulfate. In 2012, the proportion of treated flow was clearly higher, 265 mm of recorded total flow of 345 mm (77 %).

**Fig. 3:**
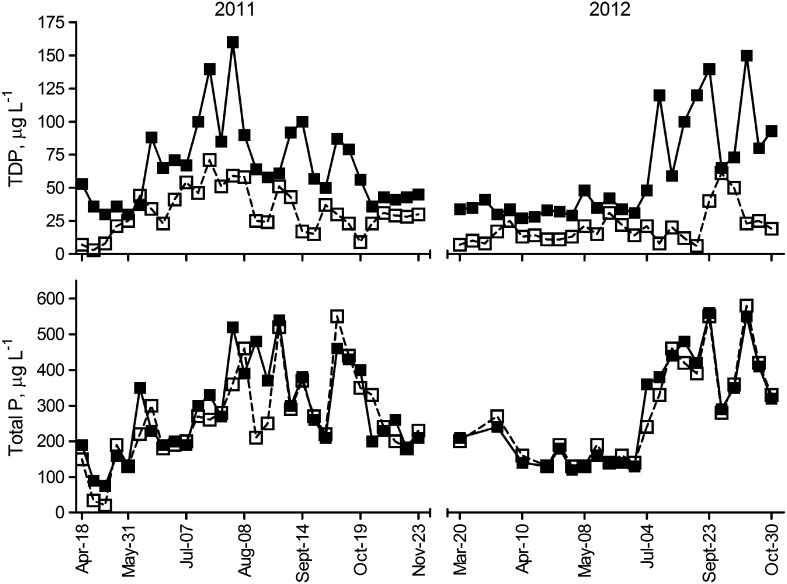
Concentrations of total dissolved P (TDP) (*upper graph*, *n* = 56) and total P (*lower graph*, *n* = 52) in water samples taken during 2011 and 2012 upstream (*closed symbols*, *solid line*) and downstream (*open symbols*, *dotted line*) of the ferric sulfate dispenser station at Nautela

Most of the total P transported through the Nautela ditch was in particulate form. Dissolved P (TDP) constituted on average 24 % of the total P in the inflow water samples. The TDP concentration varied from 0.027 to 0.160 mg L^−1^ and the total P concentration from 0.075 to 0.560 mg L^−1^ (Fig. 3). The largest TDP reductions took place at times when TDP concentrations were highest (Fig. 3), but there was no close correlation between TDP measured upstream of the dispenser unit and percentage reduction in TDP as a result of chemical application (data not shown).

During the periods when ferric sulfate was administered to water in 2011, the TDP flux was 8.2 kg and the TP flux 37 kg. In 2012, the estimated TDP flux was16 kg and the TP flux 71 kg during the treatment period. Of these TDP masses, 5.8 kg in 2011 and 13 kg in 2012 were transformed to iron-associated form, giving flow-weighted efficiency of TDP conversion of 71 and 82 % for 2011 and 2012, respectively. The arithmetic mean reduction in TDP, calculated from the results of individual water sample pairs, was 52 % (Table 2).

**Table 2:** Average dissolved P concentration upstream of the dispenser unit, (unweighted) mean reduction in dissolved P due to ferric sulfate application and (per kg dissolved P) chemical use and estimated cost of the treatment at the 15 study sites

Site	Dissolved P concentration mg L^−1^	Mean reduction in dissolved P conc. %	Ferric sulfate use in kg per kg dissolved P converted	Costs in EUR per kg dissolved P converted
Nautela	0.064	54	211	134^a^
N1	0.069	79	583	204
N2	0.062	33	243	85
N3	1.20	62	254	89
N4	0.071	78	909	318
N5	0.65	67	394	138
N6	0.020	77	953	334
N7	0.021	75	687	240
N8	1.10	79	39	14
N9	0.032	57	1241	434
N10	0.36	>95	71	25
T1	4.01	86	45	16
P1	0.061	61	417	146
P2	0.069	81	473	165
P3	0.154	79	216	76
All sites (95 % CI)		66 (61–70)	448 (248–650)	157 (87–228)^b^

^a^At Nautela, the ferric sulfate cost 0.46 EUR kg^−1^ in 2011 and 0.55 EUR kg^−1^ in 2012; at the other sites it cost 0.35 EUR kg^−1^

^b^Calculated with the 0.35 EUR kg^−1^ ferric sulfate price

Total P was unaffected by chemical application (Fig. 3). This is because the P-containing iron precipitates were small in size, did not readily form flocs in turbulent flow, and therefore did not settle in the ditch section between the dispenser unit and the sampling point. Only under minimal flow conditions was occasional floc formation observed in calm water downstream of the sampling point.

**Fig. 4:**
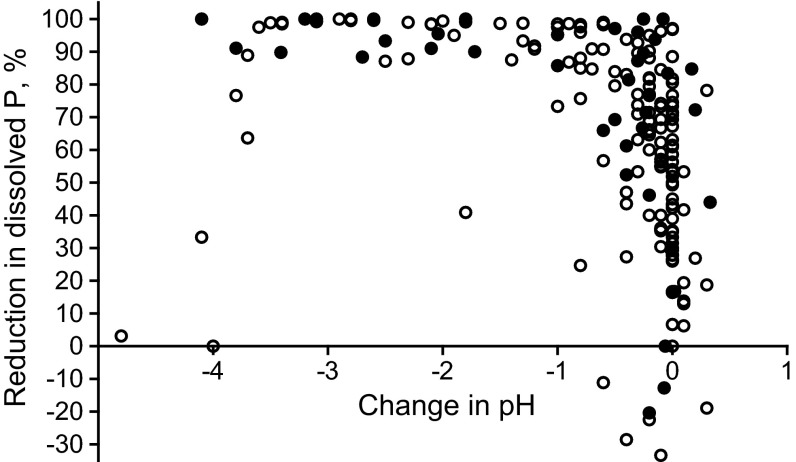
Reduction in dissolved P concentration (as a result of ferric sulfate application) as a function of pH change in water (from about neutral pH at all sites). The graph shows results of all sites and samplings; *closed symbols* for DRP and *open symbols* for TDP. Reduction based on concentration difference in samples taken upstream and downstream of dispenser units

The chemical dose applied varied according to the flow, and ranged from zero at minimal flows to tens of kg per hour in the peak flow period. The decrease in pH at lower to moderate flow volumes was about 0.5 units or less (pH in inflow 7.2 on average; data not shown), but high flows were associated with an up to 2.5 unit decrease in pH, indicating relatively higher doses of ferric sulfate in high flow regimes. The change in pH as a result of chemical administration was related to P conversion efficiency, so that a distinct decline in pH (higher chemical dose) was accompanied by higher conversion of dissolved P until the pH reached about 4, after which the conversion efficiency no longer increased, but started to decline. This relationship is shown in Fig. 4, which displays the results for all individual samples (all study sites included; at all sites pH of inflow water was about neutral).

Occasional steeper declines in pH were apparent in the pH sensor data (not shown). One sharp decline was caused by collapse of the dispenser unit platform, whereupon the hopper emptied into the ditch and the remaining chemical (more than 200 kg) dissolved in water, which was acidified to pH < 3. This acidic pulse passed in 4 h, after which pH below the dispenser station returned to normal levels. Some less dramatic acidification events (to about pH 5) took place at minimal flows in 2011, as a result of chemical slowly but continuously dissolving in nearly standing water that gradually became acidic.

In both years, annual consumption of ferric sulfate at Nautela was about 2500 kg yr^−1^. During 2011, when the chemical cost was 0.47 EUR kg^−1^ (0 % VAT, transport not included), the estimated cost of binding 1 kg dissolved P in iron-associated form at Nautela was EUR 207. In 2012, despite the price of Ferix-3 increasing to 0.55 EUR kg^−1^, the calculated cost for 1 kg precipitated TDP halved, to EUR 103. The total cost of the chemical for the 2 years was EUR 2550 and the average cost of converting the 19 kg of TDP to iron-bound form was thus 134 EUR kg^−1^ (Table 2).

### Other test sites

**Fig. 5:**
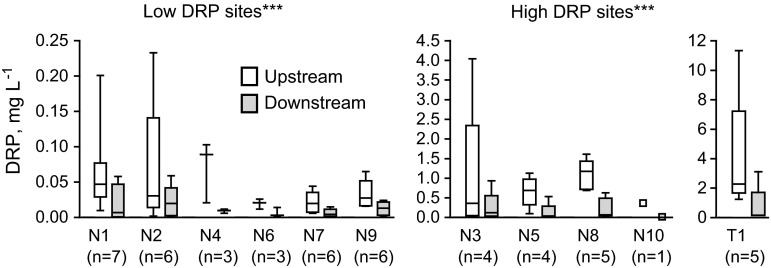
Dissolved reactive P (DRP) concentration in water sampled upstream and downstream of the ferric sulfate dispenser unit at Nuutajärvi and Tammela; *p* values refer to the difference in median concentrations within the Low DRP and High DRP groups (*n* number of sample pairs obtained at each site); *p* value: ***<0.001

**Fig. 6:**
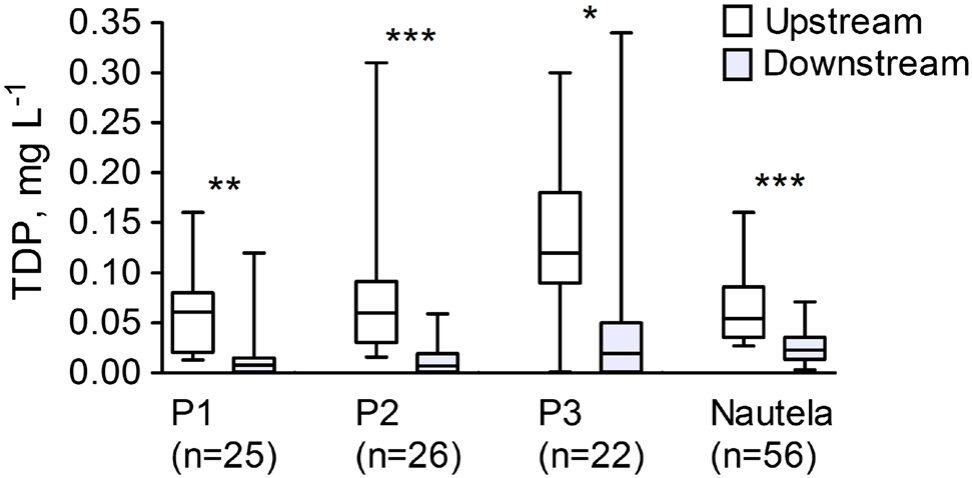
Total dissolved P (TDP) concentration in water sampled upstream and downstream of the ferric sulfate dispenser units in the river Paimionjoki area (P1–P3) and at the Nautela site; *p* value: ***<0.001, **<0.005, *<0.01 (*n* number of sample pairs obtained at each site)

Ten of the other 14 dispenser units (N1–N10) were operated during spring flows only and the remaining four units (T1, P1–P3) during spring flows and autumn flows after major rain events. The sites had highly variable dissolved P concentrations in individual samples, from non-detectable to over 11 mg L^−1^ (Figs. 5, 6). Because of the variation in catchment size, flow duration and water volumes also varied over a wide range. Dissolved P (DRP or TDP) constituted 4–90 % of the total P concentration, with substantial variation not only between sites, but also between sampling occasions at a given site.

Percentage conversion rate of dissolved P in iron-associated form at the other 14 sites was similar to that at the Nautela site, on average 60–70 % (Table 2; Fig. 5). The average percentage conversion of dissolved P was not clearly correlated with average dissolved P concentration in water (Table 2). However, there was some tendency for higher conversion efficiency at higher dissolved P concentrations when the individual sample pairs from each site were compared (data not shown).

Because flow at sites other than Nautela was recorded only in conjunction with sampling visits, estimates of the mass of P converted to less algal-available form are uncertain and the costs associated with P conversion stated in Table 2 are indicative at best. Most of these cost estimates are similar in magnitude to that for Nautela, but some sites (N8, N10, T1) showed very low costs (EUR 14–25) for binding 1 kg dissolved P to iron. These sites were characterized by small catchment area and high concentrations of dissolved P. For example, at site T1 (estimated conversion cost 16 EUR kg^−1^ dissolved P bound), the concentrations of DRP were higher than 1 mg L^−1^ during all samplings and the sample with the highest DRP, over 11 mg L^−1^, was dark brown in color and foaming, which indicated flooding of the manure pit and/or the silage bay near the upstream reaches of the ditch.

As observed at the Nautela site, some complications arose with use of the dispenser. There were two occurrences of a partly submerged dispenser unit as a result of very rapid snowmelt that flooded over the ditch banks and caused water to rise into the dispenser. This caused clumping of the chemical granules inside the feed pipe. Because ferric sulfate is hygroscopic, some clumping also occurred in damp weather or when rainwater penetrated into the hopper. In some cases, hardened ferric sulfate clumps could be only removed from the dispensers by hammering. Overdosing events also took place at some sites when ice and snow blocked the v-notch weir during frosty nights and continuous dissolution of the chemical to standing water acidified the water, sometimes to less than pH 3.

## Discussion

This study explored the potential of a relatively easily adoptable method for dispensing precipitation chemical to stream water in order to decrease the availability to water organisms of dissolved P in agricultural runoff. Precipitation chemicals can be administered to water directly, which makes them a potential way of targeting snowmelt runoff, which is a major carrier of P to watercourses in agricultural regions at northerly latitudes (Rekolainen 1989). The early phases of snowmelt in particular may contain very high dissolved P concentrations as a result of P release from grass vegetation and surface soil (Rekolainen 1989; Uusi-Kämppa 2005). These intensive runoff peaks usually take place before the soil thaws, at which time methods such as vegetated buffers, wetlands, buried or edge-of-field P traps and traditional soil management-based P mitigation measures can be expected to perform poorly.

Ferric sulfate was applied at doses designed to bring about a notable decrease in dissolved P concentration, with the actual amount being based on previous tests (Närvänen et al. 2008). Addition of the chemical did not remove P from the water, but converted it to an iron-associated form that is presumably sparingly available to microbes and algae (Li and Brett 2013). This could suppress the growth of algae and other biomass in the receiving waters, and enhance the settling of P bound in Fe–P associations to sediments in ditches, streams or in lakes. However, these Fe–P associations remain in the aquatic system and may be subjected to re-dissolution over time, for example as a result of sediment anoxia.

There were no significant changes in total P concentrations in the paired samples taken from water upstream and downstream of the dispenser units. This is because floc formation of the solid matter (and the associated particulate P) did not effectively take place with the relatively small ferric sulfate additions used. Moreover, there was no substantial accumulation of iron sludge below the sampling points downstream of the dispenser units at any of the study sites. If removal of particulate P is desired the dose should be higher, and the system would need to be optimized for that by adjusting pH, mixing and settling, sludge removal, etc. All this is relatively easily arranged in waterworks and wastewater treatment plants, but it would be a challenge to achieve in ditches at field margins. Sedimentation ponds could be used to collect sludge, but utilization of the precipitated P would then be a challenge, because P stripped by metal salts is considered uneconomic to recover for recycling (de-Bashan and Bashan 2004).

From an environmental point of view, any P entering waters should be bound in associations that have a low bioavailability, rather than being present in dissolved form that triggers growth of algae and bacteria. Overall, the results obtained at most of the test sites used in this study indicated a marked reduction, of on average about 60–70 %, in dissolved P by the ferric sulfate dispenser method. Compared with the ability of established P mitigation measures (e.g., conservation tillage, buffer zones, constructed wetlands) to decrease dissolved P concentrations, this is an impressive reduction and occurs immediately. Effects of similar magnitude have been reported in studies on P retention media (McDowell et al. 2007; Penn et al. 2007; Kirkkala et al. 2012; Penn et al. 2012), but such P barriers would still be frozen during the start of spring flow peak in the conditions of the present study.

Despite the high effectiveness displayed, the ferric sulfate dispenser method should be selectively used, in the first instance at sites identified by previous surveys as having high dissolved P concentrations. The P concentrations occurring in the water have a direct effect on the economics of chemical use. In the present study, the estimated cost of conversion of 1 kg dissolved P to a less bioavailable form ranged from approximately EUR 20 to EUR 400. These cost estimates can be taken as indicative, as the assessment of P fluxes was uncertain. If correct, the lower end of these estimates is similar to the average P abatement cost for wastewater treatment plants reported by Hautakangas et al. (2014) in their analysis of 182 treatment plants in the Baltic Sea region (EUR 15–20 kg^−1^ for an abatement target of 40–80 % of total P load). Another P mitigation measure that is comparatively low cost is gypsum application. Iho and Laukkanen (2012) calculated the annual costs of applying about 4 Mg gypsum per hectare to a field in south-west Finland based on data in a catchment study by Ekholm et al. (2012) and concluded that at an annual price of EUR 73, the field hectare would achieve the full P mitigation potential from gypsum use, i.e., about 65 % reduction in total P loss. A total P load of 2 kg ha^−1^ can be expected from a high-P soil and thus gypsum amendment would cost about EUR 50–60 kg^−1^ total P. Iho and Laukkanen (2012) related their results to the estimated marginal damage from P losses (150 EUR kg^−1^ P; Kosenius 2010), which is close to the average abatement cost estimated e.g., for Nautela. The upper end of the cost estimates in the present study (~400 EUR kg^−1^) agree with those reported by McDowell and Nash (2012) for in-field and edge-of-field measures targeting dissolved P in Australia and New Zealand.

With regard to practical applicability, issues with ferric sulfate dosing were apparent at some test sites in this study. The effect of the platform failure at the Nautela site, resulting in a very acidic pulse of water, was potentially serious. Dissolution of ferric sulfate acidifies water, i.e., leads to a drop in pH, because its reaction with water results in formation of ferric hydroxides and the liberation of protons from water molecules in the reaction: Fe_2_SO_4_ + 4H_2_O → 2FeOOH + 6H^+^ + SO_4_^2−^. The target pH change in the present study was 0.5–1.0 pH unit, and this or a smaller change was generally achieved most of the time, but some exceptions were recorded. At Nautela, the chemical hopper was rather small in relation to peak flow volumes and heavy rain occasionally emptied the dispenser. Having a small chemical hopper may be regarded as a safety precaution, but the downside is the burden of refilling the unit more frequently.

Apart from the collapse of the dispenser station at Nautela, at some other sites snow and ice hindered water flow over the v-notch weir and caused any ferric sulfate left in the dispenser to dissolve in the small volume of ponded water created during the rise in water level above the weir, resulting in a distinct drop in pH. These incidents also indicate that small ditches should be preferred over larger streams, because any possible overdosing will only have a local impact on the small ditch and the water would be diluted when it joined a larger stream.

Other difficulties in the operation of the units arose from the hygroscopic nature of the granulated chemical during periods with high air moisture, typically in autumn. A combination of low flow and damp air caused ferric sulfate clumping that in some cases blocked the doser outlet. This problem was discussed with the manufacturer of Ferix-3, which has tested different types of coating materials to overcome problems associated with hygroscopicity, but currently there seems to be no appropriate coating materials available. Coating of the chemical grains would also increase the price, which is an important criterion in chemical procurement by the main users, i.e., water and wastewater treatment plants.

## Conclusions

While not a permanent or widely applicable solution for reducing dissolved P concentrations in stream water, the ferric sulfate dispenser tested here can be an aid in P loss mitigation in the landscape. At sites characterized by high concentrations of dissolved P, relatively modest amounts of metal salts can convert dissolved P into a less available form for algae. The higher the dissolved P concentration in water and the more limited the source area, the better the cost-effectiveness of the chemical in strictly stripping P. However, for treatment of large volumes of waters with low P concentrations the ferric sulfate dispenser method is clearly too expensive to be a viable mitigation option. When ferric sulfate was dispensed to water a slight pH decline was achieved (0.5 pH units), which was found to be associated with the precipitation of dissolved P. A higher dose would also floc particulate P from the water, but would result in formation of large volumes of sludge and substantially lower pH values downstream of the dispenser unit. Moreover, with any failure of the dispenser unit the pH drop could be dramatic. Therefore, ferric sulfate dispensers would require monitoring when used in open systems such as field ditches.
